# Excitatory/inhibitory imbalance in autism: the role of glutamate and GABA gene-sets in symptoms and cortical brain structure

**DOI:** 10.1038/s41398-023-02317-5

**Published:** 2023-01-21

**Authors:** Viola Hollestein, Geert Poelmans, Natalie J. Forde, Christian F. Beckmann, Christine Ecker, Caroline Mann, Tim Schäfer, Carolin Moessnang, Sarah Baumeister, Tobias Banaschewski, Thomas Bourgeron, Eva Loth, Flavio Dell’Acqua, Declan G. M. Murphy, Nicolaas A. Puts, Julian Tillmann, Tony Charman, Emily J. H. Jones, Luke Mason, Sara Ambrosino, Rosemary Holt, Sven Bölte, Jan K. Buitelaar, Jilly Naaijen

**Affiliations:** 1 Department of Cognitive Neuroscience, Donders Institute for Brain, Cognition and Behaviour, Radboud University Medical Center, Nijmegen, the Netherlands; 2 Department of Human Genetics, Radboud University Medical Center, Nijmegen, The Netherlands; 3 Department of Psychology, Institute of Psychiatry, Psychology & Neuroscience, King’s College London, London, UK; 4 Department of Child and Adolescent Psychiatry, Psychosomatics and Psychotherapy, University Hospital Frankfurt am Main, Goethe University, Frankfurt, Germany; 5 Department of Psychiatry and Psychotherapy, Central Institute of Mental Health, University of Heidelberg, Mannheim, Germany; 6 Child and Adolescent Psychiatry, Central Institute of Mental Health, Medical Faculty Mannheim, University of Heidelberg, Mannheim, Germany; 7 Institut Pasteur, Human Genetics and Cognitive Functions Unit, Paris, France; 8 Sackler Institute for Translational Neurodevelopment, Institute of Psychiatry, Psychology & Neuroscience, King’s College London, London, UK; 9 Department of Forensic and Neurodevelopmental Sciences, Institute of Psychiatry, Psychology & Neuroscience, King’s College London, London, UK; 10 Medical Research Council (MRC) Centre for Neurodevelopmental Disorders, King’s College London, London, UK; 11 Roche Pharma Research and Early Development, Neuroscience and Rare Diseases, Roche Innovation Center Basel, F. Hoffmann–La Roche Ltd., Basel, Switzerland; 12 Department of Psychology, Institute of Psychiatry, Psychology, & Neuroscience, King’s College London, London, UK; 13 Centre for Brain and Cognitive Development, Birkbeck, University of London, Henry Wellcome Building, London, UK; 14 Department of Psychiatry, University Medical Center Utrecht Brain Center, Utrecht University, Utrecht, the Netherlands; 15 Autism Research Centre, Department of Psychiatry, University of Cambridge, Cambridge, UK; 16 Center of Neurodevelopmental Disorders (KIND), Centre for Psychiatry Research; Department of Women’s and Children’s Health, Karolinska Institutet & Stockholm Health Care Services, Region Stockholm, Stockholm, Sweden; 17 Child and Adolescent Psychiatry, Stockholm Health Care Services, Region Stockholm, Stockholm, Sweden; 18 Curtin Autism Research Group, Curtin School of Allied Health, Curtin University, Perth, Western Australia; 19 Karakter Child and Adolescent Psychiatry University Center, Nijmegen, the Netherlands

**Keywords:** Neuroscience, Biomarkers, Autism spectrum disorders

## Abstract

The excitatory/inhibitory (E/I) imbalance hypothesis posits that imbalance between excitatory (glutamatergic) and inhibitory (GABAergic) mechanisms underlies the behavioral characteristics of autism. However, how E/I imbalance arises and how it may differ across autism symptomatology and brain regions is not well understood. We used innovative analysis methods—combining competitive gene-set analysis and gene-expression profiles in relation to cortical thickness (CT) to investigate relationships between genetic variance, brain structure and autism symptomatology of participants from the AIMS-2-TRIALS LEAP cohort (autism = 359, male/female = 258/101; neurotypical control participants = 279, male/female = 178/101) aged 6–30 years. Using competitive gene-set analyses, we investigated whether aggregated genetic variation in glutamate and GABA gene-sets could be associated with behavioral measures of autism symptoms and brain structural variation. Further, using the same gene-sets, we corelated expression profiles throughout the cortex with differences in CT between autistic and neurotypical control participants, as well as in separate sensory subgroups. The glutamate gene-set was associated with all autism symptom severity scores on the Autism Diagnostic Observation Schedule-2 (ADOS-2) and the Autism Diagnostic Interview-Revised (ADI-R) within the autistic group. In adolescents and adults, brain regions with greater gene-expression of glutamate and GABA genes showed greater differences in CT between autistic and neurotypical control participants although in opposing directions. Additionally, the gene expression profiles were associated with CT profiles in separate sensory subgroups. Our results suggest complex relationships between E/I related genetics and autism symptom profiles as well as brain structure alterations, where there may be differential roles for glutamate and GABA.

## Introduction

Autism spectrum disorder (autism) is a neurodevelopmental condition characterized by challenges in social interaction and communication, restricted and repetitive patterns of behavior and/or atypical sensory processing [1]. One influential hypothesis regarding its underlying mechanisms is the excitatory/inhibitory (E/I) imbalance hypothesis, which suggests that an imbalance between excitatory (predominantly glutamatergic) and inhibitory (predominantly GABAergic (γ-aminobutyric acid)) mechanisms in the brain underlies symptomatology [2]. Causal links have been suggested, but so far with suggestions for both overexcitation and overinhibition [2–6]. However, understanding the mechanisms of *how* E/I imbalance is underlying autism symptomatology is complex. The heterogeneity and polygenic nature of autism, and previous opposing findings of E/I imbalance, may be evidence of differential involvement across autism characteristics or brain regions.

Mechanisms of E/I imbalance may have genetic underpinnings. Autism is a polygenic condition where several genetic variants together give rise to the expression of the phenotype. Progress in identifying common genetic variants associated with autism have included genes encoding proteins involved in glutamate and GABA receptors and transporters [7–10]. De novo mutations are also known to underlie a significant portion of the prevalence of autism, where additional links between genes involved in excitatory and inhibitory signaling have been found [11]. Several studies have suggested glutamatergic and GABAergic genetic links to behavioral autism phenotypes [3, 4, 12–14]. These phenotypes have been linked to changes in glutamate and GABA concentrations in the brain as well [4, 15].

Genetic and behavioral changes in autistic individuals can additionally be linked to brain structure, where a role for E/I imbalance seems plausible. Differences in cortical thickness (CT) have consistently been found in autism, and have been shown to differ throughout development as well [16, 17]. More specifically, both increased and decreased cortical thickness has been found in autism mainly in fronto-temporal, fronto-parietal, limbic areas and fronto-striatal circuits [16–22]. However, we do not yet have a clear understanding of what is causing these differences, although there is strong evidence that genetic factors play a role [21]. Cell-type specific gene-expression has been shown to be associated with differences in cortical thickness in several neurodevelopmental disorders, among which autism [23, 24]. Some genes within these cell-type specific gene-sets relate to cellular E/I function, but a similar relation has not yet been investigated focusing specifically on genetic pathways involved in excitatory and inhibitory signaling. Although not yet investigated directly, it is plausible that alterations in glutamate and GABA functioning relate to morphological differences such as CT. For instance, glutamate and GABA receptors play a role in dendritic growth, a process with genetic underpinnings found to be altered in autism [25–27]. Dendrite growth is also linked to cortical thickness [23, 28]. Altered dendritic growth, and associated genes, have been linked to autism symptomatology, especially repetitive behaviors [25, 29, 30]. In order to understand mechanistic underpinnings of morphological differences in autism it is important to get a better understanding of these links between E/I imbalance and how it may relate to structural differences. This has potential to increase understanding of the links between molecular and genetic mechanisms of autism with macroscopic measures such as cortical thickness, aiding in developing markers for subtyping and developing targeted treatment options in autism [19].

In the current study we want to integrate parts of the E/I puzzle by taking a multimodal approach focusing on aggregated (common) genetic variation, different autism phenotypes and their association with brain structure. One relatively understudied part of the autism phenotype comprises sensory symptoms. These are especially interesting as they have been suggested promising in an attempt to unscramble the autism heterogeneity [31] as well as for their shown link with E/I imbalance using MR spectroscopy [32]. Additionally, previous investigations within our dataset have shown differences in CT between those with severe and low sensory processing deficits in brain regions enriched for genes that are expressed in excitatory neurons in the developing cortex [19].

Here we used a competitive gene-set approach [33–35], investigating the role of aggregated genetic variation in glutamate and GABA gene-sets in behavioral autism phenotypes and cortical thickness. By considering several (common) genetic variants in the same analysis, the power of the study in explaining phenotypic variance is increased. In short, this tests whether genes in the gene-set are more strongly correlated with the phenotype of interest than other genes [33]. This method has shown utility in other neurodevelopmental disorders showing aggregated genetic effects rather than using single candidate-gene associations [29, 36, 37]. Additionally, using the same gene-sets, we investigated whether their expression profiles across the cortex could be associated with differences in cortical thickness between autistics and neurotypical controls (NTC). Building on the previous findings focusing on sensory symptoms [18, 31], we further extended these analyses with linking this E/I related gene-expression to cortical thickness profiles in separate sensory subgroups.

By integrating these approaches, we can deepen our understanding of the links between aggregated genetic variation in glutamate and GABA pathway signaling sets and different behavioral autism phenotypes as well as brain structure. Based on previous findings regarding excitatory or inhibitory alterations in autism, we expect to find differential involvement of the glutamate and GABA genes across the autism phenotypes, reflected in the competitive gene-set analysis. This may be further confirmed with the exploratory analyses using gene-expression in association with structural brain differences in both the autistic versus NTC as well as in the sensory symptom subgroups.

## Methods and Materials

### Participants

We included participants from the Longitudinal European Autism Project (LEAP), part of the AIMS-2-TRIALS clinical research programme (https://www.aims-2-trials.eu/) [38–40]. Our sample consisted of 638 participants (*n* = 359 autistic and *n* = 279 neurotypical controls) for whom structural MRI data was available that passed quality control [19]. Phenotypic, genetic and brain imaging data were collected at six study centers across Europe: Institute of Psychiatry, Psychology and Neuroscience, King’s College London (IoPPN/KCL, UK), Autism Research Centre, University of Cambridge (UCAM, UK), University Medical Centre Utrecht (UMCU, Netherlands), Radboud University Nijmegen Medical Centre (RUNMC, Netherlands), Central Institute of Mental Health (CIMH, Germany), and the University Campus Bio-Medico (UCBM) in Rome, Italy.

Inclusion criteria for the autism group were an existing diagnosis of autism and an age-range between 6 and 30 years. Symptoms were additionally assessed using the Autism Diagnostic Observation Schedule Second Edition (ADOS-2; [41]) and the Autism Diagnostic Interview-Revised (ADI-R; [42]). For the NTC, exclusion criterion comprised of parent- or self-report of any psychiatric disorder. Individuals who had a normative T-score of 70 or higher on the Social Responsiveness Scale Second Edition (SRS-2) were excluded. Some individuals in the autism and NTC groups had intellectual disability (ID) (autism = 53, NTC = 25), defined as an IQ score between 40 and 74. Ethical approval was obtained through ethics committees at each study site. All participants or legal guardian (where applicable) provided written informed consent. For further details of the recruitment of participants in this study see [19, 38, 39].

### Phenotypic measures

The phenotypic measures used were part of a larger test battery (see [39]). Here we included three questionnaires focusing on the core autism symptoms; the Social Responsiveness Scale-Revised (SRS-2) [43], the Repetitive Behavior Scale-Revised (RBS-R) [44], and the Short Sensory Profile (SSP) [45]. For these questionnaires, we used self- or parent-report ratings, depending on age and diagnostic group. We additionally made use of sensory symptom subgroups used in this sample previously, created based on the SSP scores and factor mixture modeling [19, 31].

### Genotyping

Genotyping was performed at the Centre National de Recherche en Génomique Humaine (CNRGH) using the Infinium OmniExpress-24v1 BeadChip Illumina. Sample quality controls such as sex check (based on the X chromosome homozygosity rate or the median of the Log R ratio of the X and Y chromosomes), Mendelian errors (transmission errors within full trios) and Identity By State were performed using PLINK 1.90. Imputation of 17 million SNPs was performed using the 700k genotyped SNPs on the Michigan Imputation Server [46]. The HRC r1.1 2016 reference panel for a European population was used, as the majority of individuals in the LEAP cohort were from European ancestry. Only autosomes were imputed. Linkage disequilibrium-based SNP pruning was done for SNPs with a MAF > 1% and SNPs with an R2 < 0.1 in windows of 500 kb were selected. This resulted in 546 participants with genotypic data (*n* = 304 autistic, *n* = 242 NTC).

Selection of the glutamate (*n* = 72 genes) and GABA (*n* = 124 genes) gene-sets was based on Ingenuity Pathway Analysis software (http://www.ingenuity.com), a frequently updated database for genetic pathway analysis. Supplemental tables S1 and S2 show an overview of the included genes. In case of any significant associations between the aggregated genetic variation in the gene-sets and the phenotypes of interest, we explored smaller gene-sets containing genes encoding glutamate/GABA receptors and transporters specifically because of their more direct role in neurotransmitter signaling [47]. Those we refer to as glu-RT (*n* = 32) and GABA-RT (*n* = 26) and are defined in the supplemental Tables S1 and S2.

### Neuroimaging data

Structural brain images were acquired on 3 T MRI scanners at all sites, with T1-weighted MPRAGE sequence (TR = 2300 ms, TE = 2.93 ms, T1 = 900 ms, voxels size = 1.1 × 1.1 × 1.2 mm, flip angle = 9°, matrix size = 256 × 256, FOV = 270 mm, 176 slices). For a summary of scanner details and acquisition parameters at each site, see Table S3 in the supplemental information. The processing of all neuroimaging data was conducted at one site for all available data. For each image a model of the cortical surface was computed using FreeSurfer v6.0 (https://surfer.nmr.mgh.harvard.edu/), using a fully automated and validated procedure [48–51]. Subsequently, each reconstructed surface went through strict quality assessments, described in detail in [19]. This quality assessment included visual inspection of reconstruction errors by independent raters, manual editing where needed, and examination of the Euler number of each FreeSurfer surface reconstruction resulting in the conclusion that there were no differences in the complexity of the reconstructed cortical surface between participant groups. This resulted in parcellated regional CT measures for all the 638 participants included in our study, with 34 regions in each hemisphere using the Desikan-Killiany atlas [52]. A more detailed description of the processing of the cortical thickness data can be found in a previous publication (see [19]).

### Gene-expression data

Gene-expression data were acquired from post-mortem human brains from the Allen Human Brain Atlas (AHBA) [53], using data from six donors (aged 24–57 years, one female) of the left hemisphere only. These whole-brain gene-expression data are open source and can be downloaded from the Allen Institute for Brain Science; http://www.brain-map.org. For more details on how these data were obtained, see [53].

Using previously described procedures [23, 24, 54], these gene-expression data were mapped onto the 34 cortical regions defined by FreeSurfer’s Desikan-Killiany Atlas [52]. These gene-expression profiles were then used in the two-step procedure described by [55] to select the most consistent profiles for inclusion in our analyses. First, the correlations of gene-expressions to the median expression values across donors were calculated, and the genes showing consistent correlation profiles were selected (donor-to-median correlation rho >0.446). Secondly, we used data from the BrainSpan Atlas, where gene-expression data in a wide age-range of donors are available (www.brainspan.org). Donors were selected within the age range of our LEAP dataset (6–30 years), which gave us 9 donors (male/female = 5/4). We calculated correlations to the median expression values in the 11 cortical regions in the AHBA-to-FreeSurfer data that were also included in the BrainSpan Atlas, using methods as described by [24]. We then selected genes that correlated between the profiles of the two atlases higher than r = 0.52 (one-sided *test p* < 0.05), which resulted in 2293 genes available in total. The overlap with the gene-sets left 29 genes in our glutamate pathway gene-set, and 42 genes in our GABA pathway gene-set. The median expression profiles across regions for these genes constitute the interregional gene-expression profiles used in our analyses.

### Analyses

All analyses included the linear effects of age, sex, IQ and site as covariates. All tests were corrected using the false discovery rate (FDR; *q* < 0.05 was considered significant) unless otherwise described.

#### Gene-set analysis

To investigate associations between aggregated genetic variation within the glutamate and GABA gene-sets and the autism phenotypes of interest (SRS-2 total score, RBS-R total score, SSP total score, and ADOS-2 and ADI-R (the last two for the autism group only)) and cortical thickness, we performed competitive gene-set analysis using MAGMA (Multi-marker Analysis of GenoMic Annotation) software (version 1.10, [33]). The analysis was performed in two steps. First, gene-based *p*-values were calculated for each gene (excluding genes located on the X-chromosome, see supplemental tables S1 and S2) on our phenotypes of interest, using a multiple linear principal components regression using F-tests. Second, we tested the association of the set, aggregating the gene-based *p*-values using competitive analysis. This gene-set analysis is done with an intercept-only linear regression model for the gene-set, which tests whether the aggregated genetic variation of the genes in a gene-set is more strongly associated with the phenotype of interest than all other genes in the genome [33].

#### Cortical thickness and clinical phenotypes

To test associations between cortical thickness (CT) and our phenotypes of interest (SRS-2 total score, RBS-R total score, SSP total score, ADOS-2 and ADI-R), we used linear regression models in the R-software package [56]. This was done in the left hemisphere only, due to the expression profile analysis being performed only in the left hemisphere. In addition to age, sex, IQ and site we added quadratic age effects and *total* mean cortical thickness as covariates as well, as described previously in [19].

#### Expression profiles

To investigate associations between expression profiles of the glutamate and GABA gene-sets and brain structure we used correlation across interregional profiles of CT with interregional profiles of gene-expression [23, 24, 57]. Profiles of CT were created by subtracting the average CT per region in the NTC group from the average CT in the autism group, as has been done previously [54]. In order to rule out effects being caused by heterogeneity in the sample, the groups were matched for age, sex and IQ using *matchit* [58] in R-software [56] performing nearest neighbor matching, resulting in *n* = 279 participants in each group. As cortical thickness is strongly associated with age [59], we additionally decided to do these profile correlation analyses for children, adolescents and adults separately. In order to verify any of these associations, analyses were replicated using structural imaging data of CT from the multi-site open-source ABIDE database [60]. We included participants in the same age range as our own sample (6–30 years), which resulted in data from 874 participants matched for age, sex and IQ (*n* = 437 in both groups). Details on these analyses and results can be found in the supplemental information and Fig. S1. Building upon previous results from our group [19, 31] and to parse some of the autism heterogeneity, we additionally performed these gene-expression analyses with interregional CT profiles in separate sensory subgroups (low, *n* = 375; moderate, *n* = 37; severe, *n* = 37). These subgroups were defined previously [31].

The interregional expression profiles of the genes in our glutamate and GABA pathway gene-sets were then correlated with the CT-difference interregional profiles (autism minus NTC across different age-groups and CT-average interregional profiles in sensory subgroups), which provided a distribution of correlation coefficients per gene-set. The distributions of correlation coefficients between the gene-expression and CT-difference interregional profiles were then tested for significance using a resampling approach of 10,000 random samples, as described in [23, 24]. In this approach, a random set of genes of the same size as the set being tested was selected (from the 2293 available) 10,000 times with the average correlation each time being used to create a null distribution. A two-tailed significance test was used to test the gene-set of interest against the null distribution.

## Results

### Demographics

Demographic and clinical characteristics are shown in Table 1. No differences were found between the autism and NTC groups in age. The autism group had a higher female-to-male ratio compared to NTC and NTC had a higher IQ than the autism group. As expected, the autism group had significantly higher scores on the SRS-2 and RBS-R and scored lower in the SSP (where lower scores indicate higher sensory sensitivity). Information on medication use can be found in Table S4 in the supplemental information.

**Table 1:** Demographic and clinical characteristics.

		NTC (*N* = 279)		Autism (*N* = 359)		Test statistic		*p*-value
Sex, m/f		178/101		258/101		KWχ2 = 4.71		0.03
	*N*	Mean	SD	Mean	SD		df	
**Age**		17.33	5.91	17.50	5.52	*t* = 0.38	576.91	0.70
**IQ**		104.79	19.72	98.88	18.25	*t* = −3.92	617.31	<0.001
**SRS-2**	555	28.88	23.36	88.99	30.79	*t* = 26.15	551.34	<0.001
**RBS-R**	436	2.59	8.39	16.34	13.94	*t* = 12.83	423.49	<0.001
**SSP**	325	176.66	15.74	139.43	27.27	*t* = −15.60	322.22	<0.001
**ADI-R** Social	345	–	–	16.70	6.68	–	–	-
Communication	345	–	–	13.24	5.63	–	–	-
Restricted repetitive	345	–	–	4.30	2.66	–	–	
**ADOS-2** Calibrated severity	353	–	–	5.40	2.76	–	–	–
Social affect	351	–	–	6.02	2.63	–	–	–
Restrictive repetitive	351	–	–	4.62	2.71	–	–	–

*NTC* Neurotypical controls, *autism* Autism Spectrum Disorder, *SD* standard deviation, *df* degrees of freedom, *SRS-2* Social Responsiveness Scale 2nd edition, *RBS-R* Repetitive Behavior Scale-Revised, *SSP* Short Sensory Profile, *ADI-R* Autism Diagnostic Interview-Revised, *Restricted repetitive* Restrictive Repetitive Behaviors domain, *Communication* ADI Communication domain, *Social* ADI Social domain, *ADOS-2* Autism Diagnostic Observation Schedule 2nd edition, *Calibrated severity* ADOS-2 Calibrated Severity Score, *Social affect* ADOS-2 Social Affect. *KW****χ****2* Kruskal-Wallis Chi-Square. Post hoc tests were Bonferroni corrected (alpha < 0.05).

### Gene-set analysis

Aggregated genetic variation within the glutamate gene-set (*n* = 72 genes) was associated with autism symptoms as defined by significant associations with all the ADI-R and ADOS-2 subscales (all *q* < 0.05, see Table 2). Repeating these analyses in the smaller glu-RT gene-set did not give the same significant results. No associations were found for any of the questionnaire scores. Genetic variation within the GABA gene-set (*n* = 124 genes) was nominally significantly associated with sensory processing (SSP total scores; *q* = 0.07) after FDR correction. Repeating these analyses in the smaller, more specific GABA-RT set gave a similar result (*q* = 0.06), see Table 2. To investigate these trend associations further, we performed similar post-hoc association analyses with all the SSP subscales. None of these were significantly associated with the genetic variation within the GABA gene-set (all *q*-values > 0.05). For more details see Table S5 in the supplemental information. Repeating the gene-set analyses with the questionnaires in the autism group separately did not result in any significant associations.

**Table 2:** Glutamate and GABA and phenotypes competitive gene-set analysis results.

Glutamate: Pathway gene-set (*N* = 72)	BETA	*P*	P_FDR_	SE
SRS	0.075	0.247	0.247	0.109
RBS-R	0.111	0.144	0.162	0.104
SSP	0.108	0.143	0.162	0.101
Diagnosis	0.171	0.048	–	0.103
**ADI communication domain**	**0.197**	**0.028**	**0.042**	**0.103**
**ADI restricted and repetitive behaviors domain**	**0.197**	**0.028**	**0.042**	**0.103**
**ADI social domain**	**0.197**	**0.028**	**0.042**	**0.103**
**ADOS restricted and repetitive behaviors**	**0.225**	**0.014**	**0.042**	**0.103**
**ADOS social affect**	**0.225**	**0.014**	**0.042**	**0.103**
**ADOS total score**	**0.225**	**0.015**	**0.042**	**0.103**
**Glutamate: Receptors/transporters gene-set (*N*** = **31)**				
SRS	−0.016	0.538	0.559	0.170
RBS-R	−0.024	0.559	0.559	0.163
SSP	0.188	0.116	0.278	0.157
Diagnosis	0.075	0.319	–	0.161
ADI communication domain	0.077	0.315	0.406	0.160
ADI restricted and repetitive behaviors domain	0.077	0.315	0.406	0.160
ADI social domain	0.077	0.315	0.406	0.160
ADOS restricted and repetitive behaviors	0.186	0.124	0.278	0.160
ADOS social affect	0.186	0.123	0.278	0.160
ADOS total score	0.186	0.124	0.278	0.160
**GABA: Pathway gene-set (*N*** = **124)**	**BETA**	***P***	**P**_**FDR**_	**SE**
SRS	−0.050	0.721	0.728	0.085
RBS-R	−0.013	0.562	0.632	0.081
**SSP**	**0.151**	**0.028**	0.248	0.079
Diagnosis	0.040	0.311	–	0.081
ADI communication domain	0.048	0.274	0.413	0.080
ADI restricted and repetitive behaviors domain	0.048	0.275	0.413	0.080
ADI social domain	0.048	0.274	0.413	0.080
ADOS restricted and repetitive behaviors	0.037	0.321	0.413	0.080
ADOS social affect	0.037	0.321	0.413	0.080
ADOS total score	0.037	0.321	0.413	0.080
**GABA: Receptors/transporters gene-set (*N*** = **23)**				
SRS	0.102	0.318	0.358	0.215
RBS-R	−0.329	0.946	0.946	0.205
**SSP**	**0.340**	**0.022**	0.198	0.198
Diagnosis	0.133	0.256	–	0.202
ADI communication domain	0.117	0.281	0.358	0.202
ADI restricted and repetitive behaviors domain	0.117	0.281	0.358	0.202
ADI social domain	0.117	0.281	0.358	0.202
ADOS restricted and repetitive behaviors	0.105	0.302	0.358	0.202
ADOS social affect	0.105	0.301	0.358	0.202
ADOS total score	0.105	0.301	0.358	0.202

*N* number of genes in analysis. Diagnosis was indicated as a binary variable. *SRS-2* Social Responsiveness Scale, Second Edition, *RBS-R* Repetitive Behavior Scale-Revised, *SSP* Short Sensory Profile, *ADI* Autism Diagnostic Interview-Revised, *ADOS* Autism Diagnostic Observation Schedule, *P*_FDR_ *p*-value corrected using False discovery rate (FDR), SE standard error of the regression coefficient. Significant results (*p*_FDR_ < 0.05) marked in bold.

We additionally investigated gene-set association with CT in the FreeSurfer cortical regions in the left hemisphere. There were some nominally significant (uncorrected *p*-values < 0.05) associations, although none survived FDR-correction. The details of these results can be seen in supplemental Tables S6 and S7.

### Cortical thickness and phenotypes

Our group previously showed vertex-wise group differences in cortical thickness between autism and NTC in the current sample [19]. Here we did not repeat these analyses but instead focused on the continuous measures of autism symptoms using the ADI-R and ADOS-2 in the autism group and the SRS-2, RBS-R and SSP questionnaires in the entire sample.

Cortical thickness in the frontal pole was positively associated with restricted and repetitive behaviors as reflected by the RBS-R total score (*b* = 0.05, *t* = 3.33, *q* = 0.03). No other results survived multiple comparisons corrections, although nominally significant negative associations were found between all ADI-R subscales and precuneus CT (communication *q* = 0.26, social *q* = 0.19, restricted and repetitive behaviors *q* = 0.05) as well as a nominally significant positive relation between the ADOS-2 total score and the social affect subscale and CT in the insula (*q* = 0.16, *q* = 0.13, respectively).

### Gene-expression profiles

**Fig. 1:**
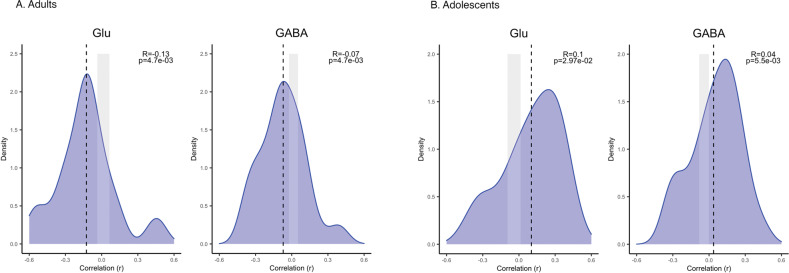
Distributions of correlation coefficients between cortical thickness difference and gene-expression. Distributions of the inter-regional correlation coefficients between differences in cortical thickness (CT) and profiles of gene-expression in adults (**A**) and adolescents (**B**). The CT-difference profiles were obtained from our LEAP data, and the expression profiles from the Allen Human Brian Atlas (AHBA), in our glutamate-pathway and GABA-pathway gene-sets. The x-axes show the correlation coefficient between CT-difference and expression profile for all genes in the gene-set; the y-axes show the estimated probability density for the correlation coefficients; the vertical dashed-lines indicates the average expression-CT difference correlation coefficient across all the marker genes in a gene-set; and the edges of the gray boxes indicates the 2.5% and 97.5%-critical values obtained from the empirical null distribution of the average expression-thickness correlation coefficient. If a vertical line sits outside the gray box, it implies that there is a significant association between gene-set and differences in CT at the unadjusted 5% significance level.

**Fig. 2:**
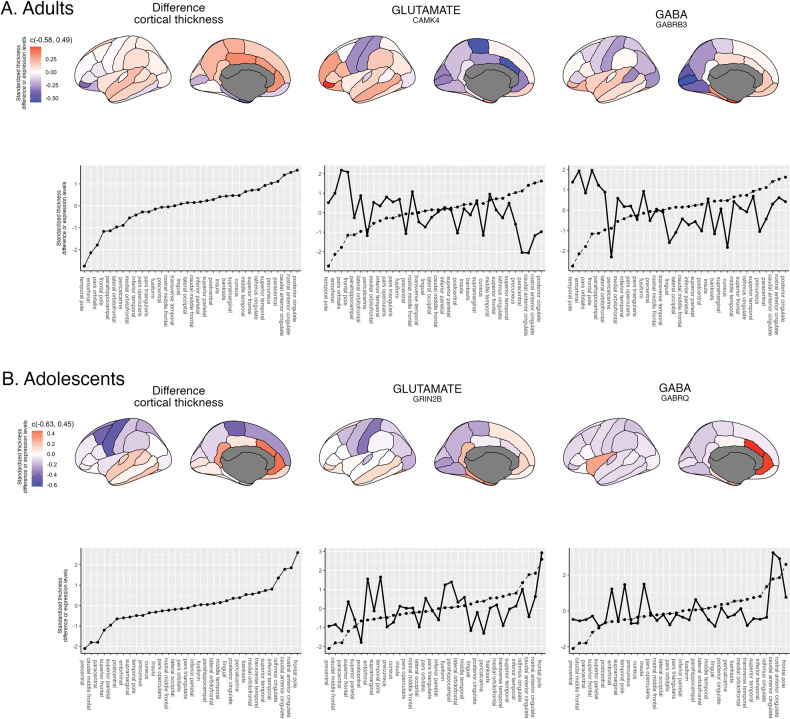
Gene-expression and cortical thickness difference from highest correlating genes. Lateral and medial views of differences in cortical thickness (CT) between autistic and neurotypical control participants in adults (**A**) and adolescents (**B**), and gene-expression levels of the genes from the glutamate and GABA (pathway) gene-sets with highest (negative in adults, positive in adolescents) correlation. Plots on the bottom row of each panel show standardized profiles of CT-differences between autistic and neurotypical control participants (dotted lines) in each age group and the gene-expression (solid lines) for the most strongly correlated gene in each respective gene-set. FreeSurfer regions on the x-axes are ordered from low to high thickness. Figures were created using ggplot2 (Ginestet, 2011) and ggseg (Mowinckel & Vidal-Piñeiro, 2020).

While the interregional profiles of group differences in cortical thickness (autism minus NTC) were not significantly associated with gene-expression profiles across our glutamate and GABA gene-sets in the full sample (all *q*-values > 0.05), splitting into groups of children, adolescents and adults gave some opposing results. In adolescents (*n* = 101 autism, *n* = 100 NTC), the interregional profile of group differences in cortical thickness was positively associated with interregional variation in expression of both glutamate (t = 2.25, *q* = 0.030, Cohen’s *d* = 0.70) and GABA genes (*t* = 3.28, *q* = 0.005, Cohen’s *d* = 1.24). In adults, on the other hand (*n* = 124 autism, *n* = 115 NTC), the group difference profile was negatively associated with expression, again for both gene-sets (glutamate: t = −2.99, *q* = 0.005, *d* = −0.93; GABA: *t* = −3.17, *q* = 0.005, *d* = −0.93), reflecting differences in CT between autistic participants and NTC changing with age. In children, no such associations were found. See Fig. 1 for the distributions of the correlation coefficients and Fig. 2 for CT differences and example genes for each gene set.

Interestingly, these results were replicated in the independent ABIDE sample for adolescents. In adults, however, positive associations between the expression profiles and CT differences were found, as opposed to the negative associations found in our LEAP sample (see supplemental information and Fig. S1).

Investigating the interregional profiles of CT in sensory subgroups separately gave positive associations with the interregional profiles of gene-expression in all groups (LOW: glutamate: *t* = 3.02, *q* = 0.004, Cohen’s *d* = 0.94; GABA: *t* = 3.19, *q* = 0.004, Cohen’s *d* = 1.21; MODERATE: glutamate: *t* = 3.18, *q* = 0.003, Cohen’s *d* = 0.99; GABA: *t* = 3.30, *q* = 0.003, Cohen’s *d* = 1.25; SEVERE: glutamate: *t* = 3.03, *q* = 0.004, Cohen’s *d* = 0.95; GABA: *t* = 3.18, *q* = 0.004, Cohen’s *d* = 1.20), see also Fig. S2. To investigate possible differences *between* the sensory subgroups we calculated interregional CT-difference profiles between groups as well, however this gave no significant associations. These results could not be replicated in ABIDE due to unavailability of the SSP questionnaire in that sample.

## Discussion

In the current study, we took a multimodal approach to investigate the role of E/I imbalance associated gene-sets in relation to behavioral phenotypes and brain structure in autism. The most important takeaway of our results is that the glutamate and GABA gene-sets were differently associated with autism symptoms, and that the expression profiles of these genes throughout the cortex were associated with differences in cortical thickness between autistic and NTC participants, depending on age. Aggregated genetic variation in the glutamate gene-set was associated with autism symptom severity on all core symptom subscales of the ADI-R and ADOS-2 (in autistic participants), while variation in the GABA gene-set showed association with sensory symptoms in the entire group, although this did not survive strict multiple comparisons correction. In adolescents and adults, but not in children, regions with greater gene-expression of glutamatergic and GABAergic genes showed greater differences in CT between autism and controls, but in opposite directions. In adolescents, this association was positive, suggesting overall higher cortical thickness in autism than in NTC, while in adults this was negative, indicating an overall higher CT in controls as opposed to autistic participants. These results provide a better understanding of the mechanistic underpinnings of the E/I imbalance hypothesis of autism, by supporting the notion that E/I imbalance varies across behavioral autism characteristics and differences in CT between autism and NTC groups.

The findings of associations between genetic variation in the glutamate gene-set and ADI-R and ADOS-2 subscale scores, and the trend associations of these subscale scores with cortical thickness in the precuneus and insula, areas known to be involved in somatosensory and visuospatial processing, interoception and self-reflection [61, 62], suggest that glutamate genes may be linked to broader autism characteristics. Additionally, the trend associations of GABA gene-sets on SSP total score and the association with cortical thickness profiles in the sensory symptom subgroups suggest a particular role for GABAergic genes in sensory processing, which supports previous findings of links between brain GABA concentrations and sensory deficits [32, 63, 64].

The lack of significant associations between aggregated variation in the glutamate and GABA gene-sets with repetitive behaviors and social responsiveness (RBS-R and SRS-2) may be considered surprising as previous studies have found links between glutamate and GABA concentrations in several brain regions and/or metabolite altering drugs with these behaviors [65–70]. However, studies investigating in vivo measures of alterations of glutamate and GABA in autism have had inconsistent results that could be due to several factors; the heterogeneity of autism, differences in study populations and brain regions investigated, or differences in processing pipelines during analysis. Furthermore, here we focused on behavioral autism characteristics and genetic information, not in vivo brain concentrations of glutamate and GABA. We did however find links between repetitive behaviors (RBS-R) and CT in the frontal pole, where increased RBS-R scores were associated with increased CT. Measures from the ADI-R and ADOS-2 diagnostic tools (in the autism group only) were differently associated with CT in the precuneus and insula, although this was only at trend-level.

We did not find direct associations of CT with SSP scores, although previous work on this dataset did find associations of differences in CT between sensory subgroups in right premotor cortex and supplementary motor areas, regions enriched for genes expressed in excitatory neurons in developing cortex [19]. In support of this, we found that regions with greater expression of genes from both gene-sets also showed greater CT in all sensory subgroups, although there were no significant differences between sensory subgroups. This show that there are likely associations of glutamatergic and GABAergic gene-expression to alterations in sensory processing but that differences may be too subtle between groups to show any differences. These associations, combined with the trend significant associations of aggregated genetic variance of the GABA gene-set are in line with previous work indicating that alterations of GABA are associated with altered sensory processing in autism [32, 71, 72]. It is also possible that we did not see significant associations with CT-difference scores between these groups due to lower number of participants on the moderate and severe groups (*n* = 37, *n* = 18 respectively).

Interregional variation in expression of glutamatergic and GABAergic genes was associated with the group differences in CT in adolescents and adults, but not in children, nor in the overall sample. Regions with greater expression of both glutamate and GABA genes showed greater differences in CT between autistic and NTC participants. These results taken together suggests possible genetic underpinnings of excitation/inhibition imbalance affecting autism symptoms. Furthermore, it suggests that there may be important differences in trajectories across development which may be mediated through altered cortical thickness. This is in line with previous work on this cohort finding differences in CT in regions enriched for genes involved in autism, where degree of deviance in CT from the NTC range correlated with increased polygenic scores for autism and symptom severity [19]. This needs to be investigated further, and future studies should preferably include measures of metabolite concentrations to draw further conclusions about these relationships.

Our results need to be interpreted with caution, as the presence of glutamate and GABA protein encoding genes does not directly translate to metabolite concentrations, and genetic alterations might not translate to a common phenotype across individuals [12]. Additionally, genes differ in coding for loss- or gain-of function, leading to reduced or increased protein function, further complicating any interpretation of *direction* of glutamate and GABA involvement in autism symptoms. However, our results strongly indicate critical roles of glutamate and GABA genes in these specific phenotypes and that the link between these measures needs to be investigated in more detail to increase our understanding of the mechanisms connecting genetics, glutamate and GABA neurotransmitters and autism symptomatology. More direct investigations of the E/I imbalance hypothesis are needed in order to investigate excitation and inhibition in vivo in relation to brain functioning. Promising new techniques combining different imaging methods, causal discovery analysis, and pharmacological interventions and longitudinal studies, will allow us to do this in the future through which we hope to further increase our understanding of how chemical imbalance in the brain is associated with functioning. Ultimately, E/I balance may be manipulated using glutamate- and/or GABA- influencing pharmacological treatments. One study already showed decreased glutamate and GABA concentrations after bumetanide treatment to be positively associated with autism symptom improvement [71].

Strengths of this study were the combination of genetic, structural and phenotypic data from the same cohort, which gave us the opportunity to for the first time analyze these data together. Another strength was the relatively large number of participants available giving us more confidence in our results. There were also some limitations. Firstly, there were fewer females than males included in this study, a common problem in autism research. Furthermore, the gene-expression data were only used in the left hemisphere. However, the gene-expression data used in the expression profile analyses was robust and only included if the interregional profiles were similar across another dataset (BrainSpan), increasing the confidence in the robustness of these profiles. Another limitation is that the AHBA donors were all neurotypical controls, and we do not know whether genes are expressed differently in autism. Additionally, there were differences in ages of participants recruited at different sites, which has been investigated in an initial analysis of the LEAP cohort [38]. We also did not fully replicate our gene-expression profile results in the independent ABIDE data set (see the supplemental information and Fig. S1). However, the results were largely overlapping showing similar effects, although in opposite direction in the adult group compared to the adults in our LEAP sample. This shows that heterogeneity of the autism sample and neurotypical controls have a large influence on the results.

In conclusion, we found that glutamate genes are associated with core behavioral autism characteristics and GABA genes may be associated with sensory processing, and that increased expression of glutamate and GABA genes are associated with larger differences in CT between autistics and NTC in adolescents and adults but not in children. This support the hypothesis that the influence of E/I imbalance varies across autism phenotypes and brain regions, suggesting that glutamate and GABA genes play different roles underlying different autism phenotypes and that this may change during development. We also showed the importance of linking structural brain measures, genetic and behavioral phenotype data together to gain a deeper understanding of possible E/I imbalance mechanisms in autism.

## Supplementary information

Supplemental material
