# Supplementary material for: Excitatory/inhibitory imbalance in autism: the role of glutamate and GABA gene-sets in symptoms and cortical brain structure
Source: Transl Psychiatry. 2023 Jan 21;13:18. doi: 10.1038/s41398-023-02317-5 (PMC9867712; doi:10.1038/s41398-023-02317-5)
Supplement: Supplementary file 1 — Supplemental material [file 41398_2023_2317_MOESM1_ESM.docx]

**Supplemental information**

**Excitatory/inhibitory imbalance in autism: the role of glutamate and GABA gene-sets in symptoms and cortical brain structure**

**Hollestein et al.**

**Table S1:** Summary table of all genes in the glutamate gene-set.

| Gene name | Entrez gene ID | Chromosome | Start position | End position | strand | NSNPS |
| --- | --- | --- | --- | --- | --- | --- |
| ABAT | 18 | 16 | 8768444 | 8878432 | + | 1013 |
| ALDH5A1 | 7915 | 6 | 24495197 | 24537435 | + | 297 |
| CALM1 | 801 | 14 | 90863327 | 90874619 | + | 47 |
| CALML5 | 51806 | 10 | 5540658 | 5541533 | - | 6 |
| CAMK4* | 814 | 5 | 110559947 | 110830584 | + | 1538 |
| DLG4 | 1742 | 17 | 7093209 | 7123369 | - | 102 |
| GAD1 | 2571 | 2 | 171673200 | 171717661 | + | 172 |
| GAD2 | 2572 | 10 | 26505236 | 26593491 | + | 579 |
| GLS | 2744 | 2 | 191745547 | 191830278 | + | 290 |
| GLUD1 | 2746 | 10 | 88809959 | 88854776 | - | 186 |
| GLUD2 | 2747 | X | 120181462 | 120183796 | + |  |
| GLUL | 2752 | 1 | 182350839 | 182361341 | - | 55 |
| GNB1 | 2782 | 1 | 1716725 | 1822552 | - | 250 |
| GNB1L | 54584 | 22 | 19775932 | 19842462 | - | 369 |
| GNB2* | 2783 | 7 | 100271363 | 100276792 | + | 19 |
| GNB3* | 2784 | 12 | 6949375 | 6956564 | + | 34 |
| GNB5* | 10681 | 15 | 52413123 | 52483565 | - | 486 |
| GNG10 | 2790 | 9 | 114423851 | 114432526 | + | 50 |
| GNG11 | 2791 | 7 | 93551016 | 93555826 | + | 32 |
| GNG12* | 55970 | 1 | 68167149 | 68299436 | - | 702 |
| GNG13 | 51764 | 16 | 848041 | 850733 | - | 33 |
| GNG2* | 54331 | 14 | 52327022 | 52436518 | + | 794 |
| GNG3 | 2785 | 11 | 62475066 | 62476678 | + | 5 |
| GNG4* | 2786 | 1 | 235710985 | 235814054 | - | 543 |
| GNG5 | 2787 | 1 | 84964006 | 84972262 | - | 37 |
| GNG7 | 2788 | 19 | 2511218 | 2702746 | - | 1041 |
| GOT1* | 2805 | 10 | 101156627 | 101190530 | - | 146 |
| GOT1L1 | 137362 | 8 | 37791799 | 37797664 | - | 17 |
| GOT2 | 2806 | 16 | 58741035 | 58768246 | - | 229 |
| **GRIA1*** | **2890** | **5** | **152870084** | **153193429** | **+** | **1819** |
| **GRIA2** | **2891** | **4** | **158141736** | **158287227** | **+** | **425** |
| **GRIA3*** | **2892** | **X** | **122317996** | **122624766** | **+** |  |
| **GRIA4** | **2893** | **11** | **105480800** | **105852819** | **+** | **1505** |
| GRID1 | 2894 | 10 | 87359312 | 88126250 | - | 4622 |
| GRID2* | 2895 | 4 | 93225453 | 94695707 | + | 7119 |
| **GRIK1** | **2897** | **21** | **30909254** | **31312282** | **-** | **2258** |
| **GRIK2*** | **2898** | **6** | **101841584** | **102517958** | **+** | **3720** |
| **GRIK3** | **2899** | **1** | **37261128** | **37499844** | **-** | **963** |
| **GRIK4*** | **2900** | **11** | **120382465** | **120859514** | **+** | **2775** |
| **GRIK5*** | **2901** | **19** | **42502468** | **42574278** | **-** | **138** |
| **GRIN1** | **2902** | **9** | **140033609** | **140063214** | **+** | **86** |
| **GRIN2A*** | **2903** | **16** | **9847265** | **10276611** | **-** | **3419** |
| **GRIN2B*** | **2904** | **12** | **13713684** | **14133022** | **-** | **2569** |
| **GRIN2C** | **2905** | **17** | **72838162** | **72856966** | **-** | **93** |
| **GRIN2D** | **2906** | **19** | **48898132** | **48948188** | **+** | **222** |
| **GRIN3A*** | **116443** | **9** | **104331634** | **104500862** | **-** | **942** |
| **GRIN3B** | **116444** | **19** | **1000437** | **1009723** | **+** | **108** |
| GRINA | 2907 | 8 | 145064226 | 145067596 | + | 9 |
| GRIP1 | 23426 | 12 | 66741178 | 67463014 | - | 4124 |
| **GRM1*** | **2911** | **6** | **146286032** | **146758782** | **+** | **2121** |
| **GRM2*** | **2912** | **3** | **51741081** | **51752629** | **+** | **16** |
| **GRM3*** | **2913** | **7** | **86273230** | **86494193** | **+** | **1110** |
| **GRM4*** | **2914** | **6** | **33989623** | **34123399** | **-** | **1020** |
| **GRM5*** | **2915** | **11** | **88237256** | **88796846** | **-** | **3817** |
| **GRM6** | **2916** | **5** | **178405328** | **178422124** | **-** | **141** |
| **GRM7*** | **2917** | **3** | **6902802** | **7783218** | **+** | **5656** |
| **GRM8*** | **2918** | **7** | **126078652** | **126892428** | **-** | **4521** |
| HOMER1 | 9456 | 5 | 78669647 | 78809659 | - | 705 |
| HOMER2 | 9455 | 15 | 83517729 | 83654905 | - | 736 |
| HOMER3 | 9454 | 19 | 19040010 | 19052041 | - | 42 |
| PICK1* | 9463 | 22 | 38453262 | 38471708 | + | 92 |
| SLC17A1 | 6568 | 6 | 25783125 | 25832287 | - | 297 |
| SLC17A2 | 10246 | 6 | 25912982 | 25930954 | - | 109 |
| **SLC17A6*** | **57084** | **11** | **22359667** | **22401049** | **+** | **208** |
| **SLC17A7*** | **57030** | **19** | **49932655** | **49945617** | **-** | **39** |
| **SLC17A8*** | **246213** | **12** | **100750857** | **100815837** | **+** | **347** |
| **SLC1A1** | **6505** | **9** | **4490427** | **4587469** | **+** | **544** |
| **SLC1A2** | **6506** | **11** | **35272752** | **35441610** | **-** | **1155** |
| **SLC1A3** | **6507** | **5** | **36606457** | **36688436** | **+** | **420** |
| SLC1A4* | 6509 | 2 | 65215579 | 65250999 | + | 145 |
| **SLC1A6** | **6511** | **19** | **15060845** | **15121455** | **-** | **503** |
| **SLC1A7** | **6512** | **1** | **53552855** | **53608304** | **-** | **472** |
| SLC38A1 | 81539 | 12 | 46576838 | 46663208 | - | 441 |
| SUCLG2 | 8801 | 3 | 67410884 | 67705038 | - | 1963 |

^All genes in table were included in the glutamate pathway gene-set. Genes marked in bold are the genes that were included in the reduced glutamate receptors/transporters gene-set (n=32). NSNPS, number of single nucleotide polymorphisms (SNPs). Two genes were excluded from the gene-set analyses (^*^GLUD2,^* *^GRIA3^*^) due to the position on the X-chromosome, resulting in n=72 genes. Genes masked with an asterisk (*) were included in the gene-expression analyses (n= 23).^

**Table S2**. Summary table of all genes in the GABA gene-set.

| Gene name | Entrez gene ID | Chromosome | Start position | End position | strand | NSNPS |
| --- | --- | --- | --- | --- | --- | --- |
| ABAT | 18 | 16 | 8768444 | 8878432 | + | 1013 |
| ADCY1* | 107 | 7 | 45614125 | 45762715 | + | 760 |
| ADCY10 | 55811 | 1 | 167778357 | 167883608 | - | 659 |
| ADCY2* | 108 | 5 | 7396343 | 7830194 | + | 2563 |
| ADCY3 | 109 | 2 | 25042038 | 25142602 | - | 694 |
| ADCY4 | 196883 | 14 | 24787555 | 24804277 | - | 81 |
| ADCY5 | 111 | 3 | 123001143 | 123167924 | - | 858 |
| ADCY6 | 112 | 12 | 49159975 | 49182820 | - | 81 |
| ADCY7* | 113 | 16 | 50278830 | 50352046 | + | 333 |
| ADCY8* | 114 | 8 | 131792546 | 132053012 | - | 1901 |
| ADCY9* | 115 | 16 | 4012650 | 4166186 | - | 1082 |
| ALDH5A1 | 7915 | 6 | 24495197 | 24537435 | + | 297 |
| ALDH9A1* | 223 | 1 | 165631449 | 165667900 | - | 239 |
| AP1B1 | 162 | 22 | 29723669 | 29784754 | - | 255 |
| AP1G2 | 8906 | 14 | 24028777 | 24038754 | - | 14 |
| AP2A1 | 160 | 19 | 50270180 | 50310369 | + | 165 |
| AP2A2 | 161 | 11 | 925809 | 1012245 | + | 487 |
| AP2B1* | 163 | 17 | 33913918 | 34053436 | + | 746 |
| AP2M1 | 1173 | 3 | 183892634 | 183901879 | + | 53 |
| AP2S1 | 1175 | 19 | 47341423 | 47354203 | - | 35 |
| CACNA1A | 773 | 19 | 13317256 | 13617274 | - | 1465 |
| CACNA1B | 774 | 9 | 140772241 | 141019076 | + | 880 |
| CACNA1C* | 775 | 12 | 2079952 | 2807115 | + | 3692 |
| CACNA1D* | 776 | 3 | 53529076 | 53847179 | + | 1844 |
| CACNA1E* | 777 | 1 | 181452447 | 181775920 | + | 1671 |
| CACNA1F* | 778 | X | 49061523 | 49089833 | - |  |
| CACNA1G* | 8913 | 17 | 48638429 | 48704835 | + | 310 |
| CACNA1H* | 8912 | 16 | 1203241 | 1271772 | + | 422 |
| CACNA1I | 8911 | 22 | 39966758 | 40085740 | + | 591 |
| CACNA1S | 779 | 1 | 201008635 | 201081694 | - | 505 |
| CACNA2D1 | 781 | 7 | 81575760 | 82073031 | - | 3150 |
| CACNA2D2* | 9254 | 3 | 50400230 | 50540892 | - | 656 |
| CACNA2D3 | 55799 | 3 | 54156620 | 55108584 | + | 5930 |
| CACNA2D4 | 93589 | 12 | 1901123 | 2027870 | - | 775 |
| CACNB1 | 782 | 17 | 37329709 | 37353956 | - | 89 |
| CACNB2* | 783 | 10 | 18429373 | 18830688 | + | 2968 |
| CACNB3 | 784 | 12 | 49208215 | 49222726 | + | 46 |
| CACNB4* | 785 | 2 | 152689285 | 152955593 | - | 1246 |
| CACNG1 | 786 | 17 | 65040652 | 65052913 | + | 56 |
| CACNG2* | 10369 | 22 | 36956916 | 37098690 | - | 720 |
| CACNG3* | 10368 | 16 | 24266874 | 24373737 | + | 675 |
| CACNG4 | 27092 | 17 | 64960980 | 65029518 | + | 432 |
| CACNG5 | 27091 | 17 | 64831235 | 64881941 | + | 373 |
| CACNG6 | 59285 | 19 | 54494403 | 54515920 | + | 115 |
| CACNG7 | 59284 | 19 | 54412704 | 54447195 | + | 105 |
| CACNG8 | 59283 | 19 | 54466290 | 54493469 | + | 111 |
| CATSPER1 | 117144 | 11 | 65784223 | 65793988 | - | 45 |
| CATSPER2 | 117155 | 15 | 43922772 | 43941039 | - | 63 |
| CATSPER3 | 347732 | 5 | 134303596 | 134347397 | + | 207 |
| CATSPER4 | 378807 | 1 | 26517119 | 26529033 | + | 107 |
| DNM1 | 1759 | 9 | 130965634 | 131017528 | + | 223 |
| **GABARAP** | **11337** | **17** | **7143738** | **7145753** | **-** | **5** |
| **GABBR1*** | **2550** | **6** | **29570005** | **29600962** | **-** | **219** |
| **GABBR2** | **9568** | **9** | **101050364** | **101471479** | **-** | **2637** |
| **GABRA1** | **2554** | **5** | **161274197** | **161326965** | **+** | **283** |
| **GABRA2*** | **2555** | **4** | **46246470** | **46392056** | **-** | **727** |
| **GABRA3*** | **2556** | **X** | **151334706** | **151619831** | **-** |  |
| **GABRA4** | **2557** | **4** | **46920917** | **46996424** | **-** | **406** |
| **GABRA5*** | **2558** | **15** | **27111866** | **27194357** | **+** | **158** |
| **GABRA6** | **2559** | **5** | **161112658** | **161129598** | **+** | **81** |
| **GABRB1*** | **2560** | **4** | **47033295** | **47432801** | **+** | **2058** |
| **GABRB2** | **2561** | **5** | **160715426** | **160975130** | **-** | **1268** |
| **GABRB3*** | **2562** | **15** | **26788693** | **27018935** | **-** | **1332** |
| **GABRD*** | **2563** | **1** | **1950768** | **1962192** | **+** | **10** |
| **GABRE*** | **2564** | **X** | **151121596** | **151143156** | **-** |  |
| **GABRG1*** | **2565** | **4** | **46037786** | **46126082** | **-** | **496** |
| **GABRG2** | **2566** | **5** | **161494648** | **161582545** | **+** | **435** |
| **GABRG3** | **2567** | **15** | **27216429** | **27778373** | **+** | **2556** |
| **GABRP** | **2568** | **5** | **170210723** | **170241051** | **+** | **193** |
| **GABRQ*** | **55879** | **X** | **151806637** | **151821825** | **+** |  |
| **GABRR1** | **2569** | **6** | **89887223** | **89941007** | **-** | **344** |
| **GABRR2** | **2570** | **6** | **89966840** | **90025018** | **-** | **405** |
| **GABRR3** | **200959** | **3** | **97705527** | **97754148** | **-** | **264** |
| GAD1 | 2571 | 2 | 171673200 | 171717661 | + | 172 |
| GAD2 | 2572 | 10 | 26505236 | 26593491 | + | 579 |
| GNA11 | 2767 | 19 | 3094408 | 3121468 | + | 144 |
| GNA12 | 2768 | 7 | 2767739 | 2883963 | - | 883 |
| GNA13 | 10672 | 17 | 63005407 | 63052920 | - | 84 |
| GNA14* | 9630 | 9 | 80037995 | 80263232 | - | 1496 |
| GNA15 | 2769 | 19 | 3136191 | 3163766 | + | 201 |
| GNAI1 | 2770 | 7 | 79764140 | 79848725 | + | 383 |
| GNAI2* | 2771 | 3 | 50264120 | 50296786 | + | 114 |
| GNAI3 | 2773 | 1 | 110091186 | 110138465 | + | 181 |
| GNAL* | 2774 | 18 | 11689014 | 11885684 | + | 1003 |
| GNAO1* | 2775 | 16 | 56225251 | 56391356 | + | 866 |
| GNAQ | 2776 | 9 | 80335189 | 80646219 | - | 1344 |
| GNAS | 2778 | 20 | 57414756 | 57486250 | + | 323 |
| GNAT1 | 2779 | 3 | 50229043 | 50235129 | + | 12 |
| GNAT2 | 2780 | 1 | 110145889 | 110155705 | - | 45 |
| GNAZ | 2781 | 22 | 23412669 | 23467224 | + | 256 |
| GNB1 | 2782 | 1 | 1716725 | 1822552 | - | 250 |
| GNB1L | 54584 | 22 | 19775932 | 19842462 | - | 369 |
| GNB2* | 2783 | 7 | 100271363 | 100276792 | + | 19 |
| GNB3* | 2784 | 12 | 6949375 | 6956564 | + | 34 |
| GNB4* | 59345 | 3 | 179113876 | 179169371 | - | 290 |
| GNB5* | 10681 | 15 | 52413123 | 52483565 | - | 486 |
| GNG10 | 2790 | 9 | 114423851 | 114432526 | + | 50 |
| GNG11 | 2791 | 7 | 93551016 | 93555826 | + | 32 |
| GNG12* | 55970 | 1 | 68167149 | 68299436 | - | 702 |
| GNG13 | 51764 | 16 | 848041 | 850733 | - | 33 |
| GNG2* | 54331 | 14 | 52327022 | 52436518 | + | 794 |
| GNG3 | 2785 | 11 | 62475066 | 62476678 | + | 5 |
| GNG4* | 2786 | 1 | 235710985 | 235814054 | - | 543 |
| GNG5 | 2787 | 1 | 84964006 | 84972262 | - | 37 |
| GNG7 | 2788 | 19 | 2511218 | 2702746 | - | 1041 |
| GPHN | 10243 | 14 | 66974125 | 67648525 | + | 3011 |
| GPR37 | 2861 | 7 | 124385655 | 124406079 | - | 81 |
| KCNH2 | 3757 | 7 | 150642044 | 150675402 | - | 179 |
| KCNN1 | 3780 | 19 | 18062111 | 18110133 | + | 207 |
| KCNN2* | 3781 | 5 | 113698016 | 113832197 | + | 840 |
| KCNN3* | 3782 | 1 | 154669938 | 154842754 | - | 925 |
| KCNN4 | 3783 | 19 | 44270685 | 44286269 | - | 72 |
| KCNQ2 | 3785 | 20 | 62031561 | 62103993 | - | 607 |
| KCNQ3 | 3786 | 8 | 133133105 | 133493004 | - | 2095 |
| MRAS* | 22808 | 3 | 138066490 | 138124377 | + | 307 |
| NSF | 4905 | 17 | 44668035 | 44834830 | + | 108 |
| OPN1SW | 611 | 7 | 128412543 | 128415844 | - | 20 |
| RPS27A | 6233 | 2 | 55459039 | 55462989 | + | 27 |
| SLC32A1 | 140679 | 20 | 37353105 | 37358015 | + | 20 |
| **SLC6A1** | **6529** | **3** | **11034420** | **11080935** | **+** | **267** |
| **SLC6A11** | **6538** | **3** | **10857917** | **10980146** | **+** | **739** |
| **SLC6A12** | **6539** | **12** | **299243** | **323740** | **-** | **169** |
| **SLC6A13** | **6540** | **12** | **329787** | **372039** | **-** | **322** |
| UBA52 | 7311 | 19 | 18674576 | 18688270 | + | 83 |
| UBB | 7314 | 17 | 16284367 | 16286059 | + | 7 |
| UBC | 7316 | 12 | 125396192 | 125399587 | - | 23 |
| UBD | 10537 | 6 | 29523389 | 29527702 | - | 42 |
| UBQLN1 | 29979 | 9 | 86274878 | 86323168 | - | 265 |

^All genes in table were included in the GABA pathway gene-set. Genes marked in bold are the genes that were included in the reduced GABA receptors/transporters gene-set (n=26). NSNPS, number of single nucleotide polymorphisms (SNPs). Four genes were excluded from gene-set analyses (^*^CACNA1F, GABRA3, GABRE, GABRQ)^*^) due to the position on the X-chromosome, resulting in n=128 genes. Genes masked with an asterisk (*) were included in the gene-expression analyses (n= 39).^

**Table S3:** Scanner parameters across sites

| Site | Manufacturer | Model | Software version | Acquisition sequence | Slices | TR  [s] | TE  [ms] | FA  [˚] | Coverage | Thickness  [mm] | Resolution  [mm^3^] | FOV |
| --- | --- | --- | --- | --- | --- | --- | --- | --- | --- | --- | --- | --- |
| Cambridge | Siemens | Verio | Syngo MR B17 | Tfl3d1_ns | 176 | 2.3 | 2.95 | 9 | 256*256 | 1.2 | 1.1*1.1*1.2 | 270 |
| KCL | GE Medical systems | Discovery mr750 | LX MR DV23.1_V02_1317.c | SAG ADNI GO ACC SPGR | 196 | 7.31 | 3.02 | 11 |  |  |  |  |
| Mannheim | Siemens | TimTrio | Syngo MR B17 | MPRAGE ADNI | 176 | 2.3 | 2.93 | 9 |  |  |  |  |
| Nijmegen | Siemens | Skyra | Syngo MR D13 | Tfl3d1_16ns | 176 | 2.3 | 2.93 | 9 |  |  |  |  |
| Rome | GE Medical systems | Signa HDxt | 24/LX/MR HD16.0_V02_1131.a | SAG ADNI GO ACC SPGR | 172 | 5.96 | 1.76 | 11 |  |  |  |  |
| Utrecht | Philips Medical Systems | Achieva/ Ingenia CX | 3.2.3/3.2.3.1/  5.1.9/5.1.9.1 | ADNI GO 2 | 170 | 6.76 | 3.1 | 9 |  |  |  |  |

Abbreviations: FA, flip angle; FOV, field of view; TE, echo time; TR, repetition time.

**Cortical thickness data processing and quality assessments.** All data was processed using the default FreeSurfer v6.0.0 software (<http://surfer.nmr.mgh.harvard.edu/>). The surface reconstructions were then visually inspected for reconstruction errors and rated by three independent raters, blind to group membership. After manual editing the (310) images were (re)preprocessed and visually (re)assessed. To assess the influence of the data quality on subsequent results, a previous study (1) examined the Euler number of each FreeSurfer surface reconstructions following manual editing. As the Euler number is calculated in each hemisphere, the sum of values across hemispheres were computed, creating one value per subject. They found no significant differences in the total Euler number between groups, indicating that the diagnostic groups have matching surface reconstruction quality. Additionally, covarying for the total Euler number in their initial analyses did not significantly affect the results. This shows that results are largely unaffected by the quality of surface reconstruction.

**Table S4:** Medication information

|  | **ASD** | **NTC** |
| --- | --- | --- |
| **N** | 122 | 20 |
| Antidepressants  SSRIs  Tetracyclic (TeCA)  Tricyclic (TCA) | 34  29  2  3 | 6  5  1  0 |
| Antiepileptics | 11 | 2 |
| Antimigraine preparations | 4 | 0 |
| Antipsychotics  Aripiprazole  Clozapine  Pipamperone  Quetiapine  Risperidone | 28  6  1  2  1  18 | 1  0  0  0  0  1 |
| Anxiolytics | 2 | 1 |
| Drugs used in Addictive Disorder | 0 | 1 |
| Hypnotics & Sedatives  Hyoscine butylbromide  Melatonin  Niaprazine | 40  1  38  1 | 2  0  2  0 |
| Other Analgesics & Antipyretics  Opioids  Others | 4  1  3 | 4  0  4 |
| Psychostimulants & Other drugs used to treat ADHD  Atomoxetine  Dexamfetamine  Methylphenidate hydrochloride | 47  3  1  43 | 9  2  0  7 |

Note. Participants may have taken up to 3 different types of medication across the listed categories during study participation.

**Table S5:** Glutamate and GABA and SSP subscale competitive gene-set analysis results

| **Glutamate: Pathway gene-set (N=72)** | **BETA** | **P** | | **P_FDR_** | **SE** |
| --- | --- | --- | --- | --- | --- |
| SSP Auditory filtering | 0.008 | 0.469 | | 0.729 | 0.101 |
| SSP Low energy/weak | 0.115 | 0.129 | | 0.362 | 0.101 |
| SSP Movement sensitivity | -0.082 | 0.789 | | 0.850 | 0.102 |
| SSP Tactile sensitivity | -0.021 | 0.581 | | 0.772 | 0.104 |
| SSP Taste/smell sensitivity | -0.112 | 0.867 | | 0.867 | 0.100 |
| SSP Underresponsive/seeks attention | 0.1585 | 0.061 | | 0.212 | 0.102 |
| SSP Visual/auditory sensitivity | -0.028 | 0.606 | | 0.772 | 0.102 |
| **Glutamate: Receptors/transporters gene-set (N=31)** |  |  | |  |  |
| SSP Auditory filtering | 0.096 | | 0.270 | 0.541 | 0.156 |
| SSP Low energy/weak | 0.355 | | 0.012 | 0.152 | 0.156 |
| SSP Movement sensitivity | 0.104 | | 0.254 | 0.541 | 0.158 |
| SSP Tactile sensitivity | 0.273 | | 0.045 | 0.208 | 0.161 |
| SSP Taste/smell sensitivity | 0.023 | | 0.442 | 0.729 | 0.155 |
| SSP Underresponsive/seeks attention | 0.318 | | 0.0218 | 0.152 | 0.158 |
| SSP Visual/auditory sensitivity | -0.092 | | 0.719 | 0.839 | 0.158 |
| **GABA: Pathway gene-set (N=124)** | **BETA** | | **P** | **P_FDR_** | **SE** |
| SSP Auditory filtering | -0.004 | | 0.522 | 0.905 | 0.080 |
| SSP Low energy/weak | 0.022 | | 0.391 | 0.905 | 0.080 |
| SSP Movement sensitivity | -0.029 | | 0.640 | 0.905 | 0.081 |
| SSP Tactile sensitivity | -0.076 | | 0.825 | 0.905 | 0.082 |
| SSP Taste/smell sensitivity | -0.104 | | 0.905 | 0.905 | 0.080 |
| SSP Underresponsive/seeks attention | 0.117 | | 0.075 | 0.526 | 0.081 |
| SSP Visual/auditory sensitivity | -0.049 | | 0.726 | 0.905 | 0.081 |
| **GABA: Receptors/transporters gene-set (N=23)** |  | |  |  |  |
| SSP Auditory filtering | 0.061 | | 0.383 | 0.836 | 0.206 |
| SSP Low energy/weak | -0.201 | | 0.836 | 0.836 | 0.206 |
| SSP Movement sensitivity | 0.211 | | 0.153 | 0.537 | 0.207 |
| SSP Tactile sensitivity | -0.172 | | 0.793 | 0.836 | 0.212 |
| SSP Taste/smell sensitivity | -0.083 | | 0.657 | 0.836 | 0.205 |
| SSP Underresponsive/seeks attention | 0.228 | | 0.137 | 0.537 | 0.208 |
| SSP Visual/auditory sensitivity | -0.176 | | 0.801 | 0.836 | 0.208 |

N, number of genes in analysis. Diagnosis was indicated as a binary variable. SSP, Short Sensory Profile; P_FDR_ p-value corrected using False discovery rate (FDR); SE, standard error of the regression coefficient. Significant results (p_FDR_<0.05) marked in bold.

**Competitive gene-set analysis on cortical thickness**

**Table S6**. Glutamate - left hemisphere competitive gene-set analysis

| FreeSurfer region | NGENES | BETA | *P* | *P*_FDR_ |
| --- | --- | --- | --- | --- |
| Banks of superior temporal sulcus | 72 | -0.028 | 0.603 | 0.967 |
| **Caudal anterior cingulate cortex** | **72** | **0.182** | **0.042** | 0.776 |
| Caudal middle frontal gyrus | 72 | -0.047 | 0.671 | 0.967 |
| Cuneus | 72 | -0.090 | 0.809 | 0.967 |
| Entorhinal cortex | 72 | 0.010 | 0.461 | 0.967 |
| Frontal pole | 72 | 0.030 | 0.388 | 0.967 |
| Fusiform gyrus | 72 | -0.045 | 0.669 | 0.967 |
| Inferior parietal cortex | 72 | -0.090 | 0.801 | 0.967 |
| Inferior temporal gyrus | 72 | -0.115 | 0.867 | 0.967 |
| Insula | 72 | 0.030 | 0.387 | 0.967 |
| Isthmus-cingulate cortex | 72 | -0.125 | 0.883 | 0.967 |
| Lateral occipital gyrus | 72 | -0.107 | 0.851 | 0.967 |
| Lateral orbital frontal cortex | 72 | 0.164 | 0.064 | 0.776 |
| Lingual gyrus | 72 | 0.105 | 0.157 | 0.967 |
| Medial orbital frontal cortex | 72 | 0.075 | 0.244 | 0.967 |
| Middle temporal gyrus | 72 | -0.056 | 0.698 | 0.967 |
| Paracentral lobule | 72 | 0.046 | 0.330 | 0.967 |
| Parahippocampal gyrus | 72 | -0.030 | 0.611 | 0.967 |
| Pars opercularis | 72 | -0.077 | 0.765 | 0.967 |
| Pars orbitalis | 72 | 0.157 | 0.068 | 0.776 |
| Pars triangularis | 72 | -0.054 | 0.694 | 0.967 |
| Pericalcarine cortex | 72 | -0.001 | 0.503 | 0.967 |
| Postcentral gyrus | 72 | -0.200 | 0.971 | 0.971 |
| Posterior cingulate cortex | 72 | -0.088 | 0.792 | 0.967 |
| Precentral gyrus | 72 | -0.045 | 0.664 | 0.967 |
| Precuneus cortex | 72 | -0.012 | 0.548 | 0.967 |
| Rostral anterior cingulate cortex | 72 | -0.130 | 0.888 | 0.967 |
| Rostral middle frontal gyrus | 72 | -0.038 | 0.640 | 0.967 |
| Superior frontal gyrus | 72 | -0.057 | 0.702 | 0.967 |
| Superior parietal cortex | 72 | -0.051 | 0.686 | 0.967 |
| Superior temporal gyrus | 72 | -0.160 | 0.936 | 0.967 |
| Supramarginal gyrus | 72 | -0.166 | 0.939 | 0.967 |
| Temporal pole | 72 | -0.047 | 0.667 | 0.967 |
| Transverse temporal cortex | 72 | -0.106 | 0.846 | 0.967 |

^NGENES, number of genes in analysis. Significant associations are marked in bold.^

**Table S7**. GABA - left hemisphere competitive gene-set analysis

| FreeSurfer region | NGENES | BETA | P | P_FDR_ |
| --- | --- | --- | --- | --- |
| Caudal anterior cingulate cortex | 124 | -0.021 | 0.599 | 0.956 |
| Caudal middle frontal gyrus | 124 | 0.086 | 0.150 | 0.555 |
| Cuneus | 124 | 0.066 | 0.211 | 0.652 |
| Entorhinal cortex | 124 | -0.090 | 0.867 | 0.956 |
| Frontal pole | 124 | 0.084 | 0.154 | 0.555 |
| Fusiform gyrus | 124 | 0.119 | 0.074 | 0.458 |
| Inferior parietal cortex | 124 | -0.046 | 0.716 | 0.956 |
| Inferior temporal gyrus | 124 | -0.079 | 0.829 | 0.956 |
| Insula | 124 | 0.017 | 0.415 | 0.940 |
| Isthmus-cingulate cortex | 124 | -0.101 | 0.894 | 0.956 |
| Lateral occipital gyrus | 124 | 0.036 | 0.331 | 0.812 |
| Lateral orbital frontal cortex | 124 | -0.070 | 0.806 | 0.956 |
| Lingual gyrus | 124 | 0.082 | 0.163 | 0.555 |
| Medial orbital frontal cortex | 124 | -0.078 | 0.829 | 0.956 |
| **Middle temporal gyrus** | **124** | **0.227** | **0.004** | 0.127 |
| Paracentral lobule | 124 | -0.145 | 0.956 | 0.956 |
| Parahippocampal gyrus | 124 | -0.004 | 0.519 | 0.956 |
| Pars opercularis | 124 | -0.047 | 0.715 | 0.956 |
| Pars orbitalis | 124 | 0.094 | 0.129 | 0.555 |
| Pars triangularis | 124 | 0.116 | 0.081 | 0.458 |
| **Pericalcarine cortex** | **124** | **0.172** | **0.020** | 0.347 |
| Postcentral gyrus | 124 | -0.073 | 0.813 | 0.956 |
| Posterior cingulate cortex | 124 | -0.057 | 0.755 | 0.956 |
| Precentral gyrus | 124 | 0.004 | 0.482 | 0.956 |
| Precuneus cortex | 124 | -0.078 | 0.825 | 0.956 |
| Rostral anterior cingulate cortex | 124 | -0.030 | 0.643 | 0.956 |
| Rostral middle frontal gyrus | 124 | 0.118 | 0.080 | 0.458 |
| **Superior frontal gyrus** | **124** | **0.155** | **0.031** | 0.352 |
| Superior parietal cortex | 124 | 0.036 | 0.335 | 0.812 |
| Superior temporal gyrus | 124 | -0.054 | 0.746 | 0.956 |
| Caudal anterior cingulate cortex | 124 | -0.114 | 0.918 | 0.956 |
| Supramarginal gyrus | 124 | -0.129 | 0.937 | 0.956 |
| Temporal pole | 124 | 0.049 | 0.280 | 0.793 |
| Transverse temporal cortex | 124 | -0.094 | 0.875 | 0.956 |

^NGENES, number of genes in analysis. Significant associations are marked in bold.^

**Replicating gene-expression analysis using ABIDE data.** The ABIDE cortical thickness (CT) data was acquired from the open source data base (http://fcon_1000.projects.nitrc.org/indi/abide/) (2), where we selected participants within the same age-range (6-30 years) as our LEAP sample, matched for age, sex and IQ (autism =437, male/female = 385/52, NTC =437, male/female = 349/88). To replicate the analysis performed in our LEAP sample, we calculated CT-difference scores between the autism and neurotypical control group (NTC) as described in the manuscript.

Gene expression analysis using the ABIDE CT-difference interregional profiles showed no significant associations in the whole sample. However, separating the participants into age groups (children, adolescents and adults, see also main manuscript), showed similar results to our findings in LEAP, especially for the adolescents (n=188 autism, n=181 NTC), where the same positive association between CT differences and both glutamate and GABA expression profiles were found (glutamate: t=1.94, *q*=0.059, Cohen's d=0.61; GABA: t=3.56, *q*=0.003, Cohen's d=1.34), although only nominally significant for glutamate. As opposed to the LEAP sample, also in adults (n=104 autism, n=125 NTC) a positive association was found between the interregional profile of differences in CT (autism-NTC) and profiles of glutamate and GABA gene expression (t=2.38, *q*=0.023, d=0.74 and t=2.51, *q*=0.023, d=0.95, respectively). No significant associations were found in children.


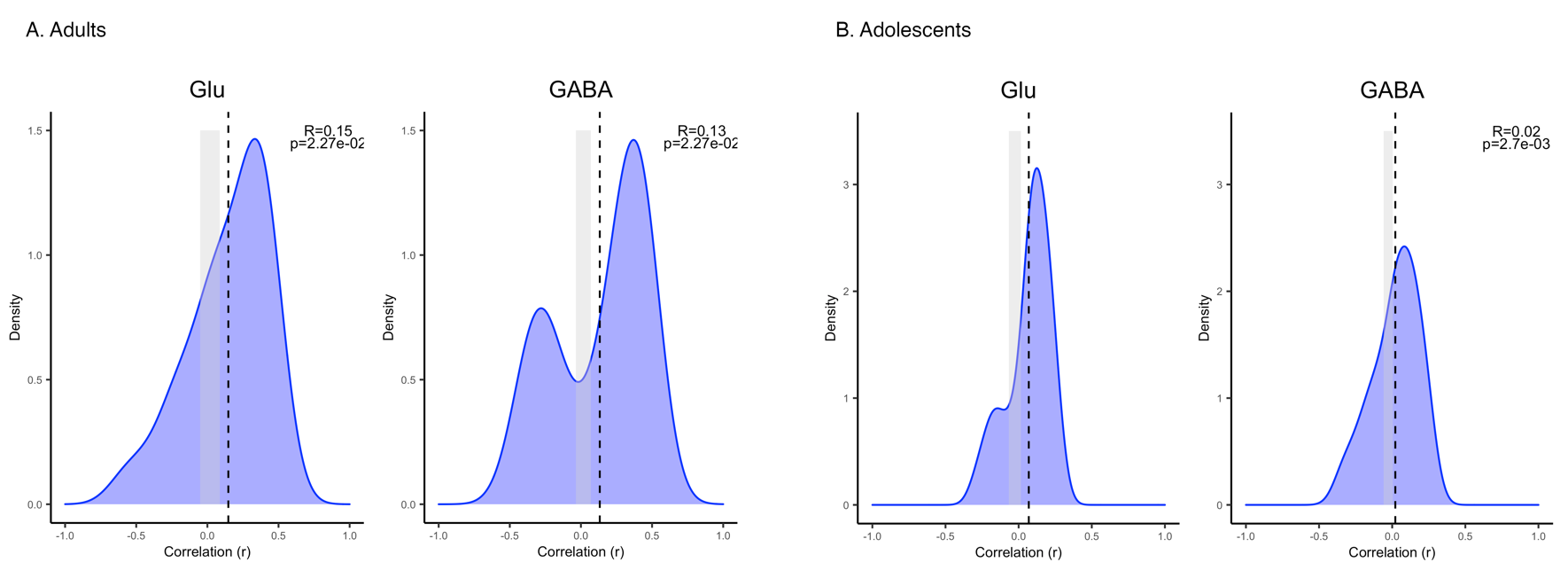


**FIGURE S1.** Distributions of the inter-regional correlation coefficients between differences in cortical thickness (CT) and profiles of gene-expression in adults (A) and adolescents (B). The CT-difference profile was obtained from the ABIDE data, and the expression profiles from the Allen Human Brian Atlas (AHBA), in our glutamate- and GABA gene-sets. The x-axes show the correlation coefficient between CT-difference and expression profile for all genes in the gene-set; the y-axes show the estimated probability density for the correlation coefficients; the vertical dashed-lines indicates the average expression-CT difference correlation coefficient across all the marker genes in a gene-set; and the edges of the gray boxes indicates the 2.5% and 97.5%-critical values obtained from the empirical null distribution of the average expression-thickness correlation coefficient. If a vertical line sits outside the gray box, it implies that there is a significant association between gene-set and differences in CT at the unadjusted 5% significance level.

**
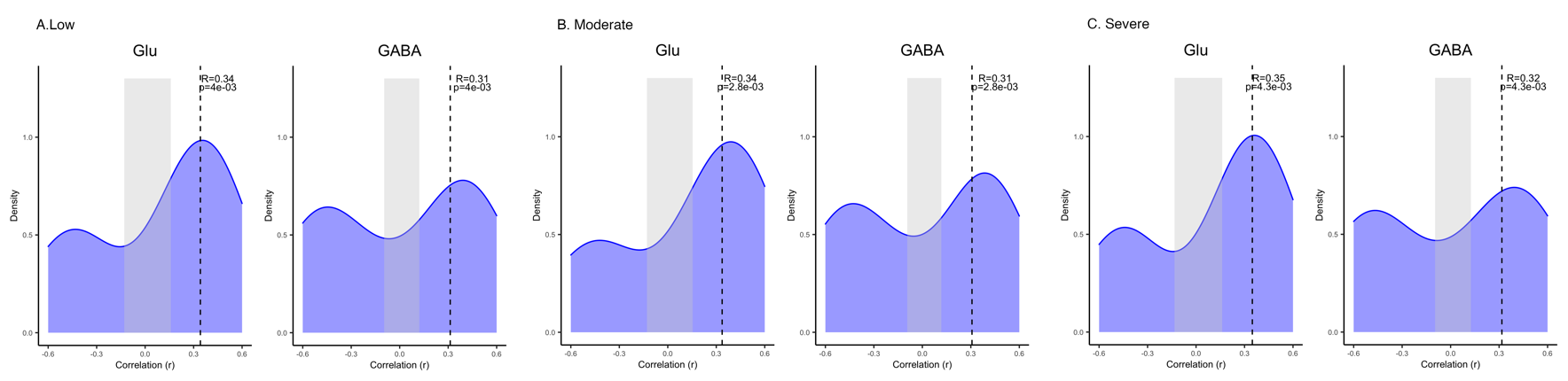
Sensory processing subgroups.** The LEAP data was separated into low, moderate or severe sensory processing subgroups (3). Interregional CT profiles were calculated by taking the average CT across participants in each brain region, in each sensory subgroup separately. Interregional CT profiles were significantly associated with both glutamate and GABA pathway gene expression in all sensory processing subgroups (LOW: glutamate: t=3.02, *q*=0.004, Cohen's d = 0.94; GABA: t=3.19, *q*=0.004, Cohen's d=1.21; MODERATE: glutamate: t=3.18, *q*=0.003, Cohen's d = 0.99; GABA: t=3.30, *q*=0.003, Cohen's d=1.25; SEVERE: glutamate: t=3.03, *q*=0.004, Cohen's d = 0.95; GABA: t=3.18, *q*=0.004, Cohen's d=1.20), see Figure S2. Regions with increased expression of glutamate and GABA genes show greater CT across all sensory subgroups.

**Figure S2.** Distributions of the inter-regional correlation coefficients between interregional profiles cortical thickness (CT) and profiles of gene-expression in separate sensory subgroups (A – sensory low, B – sensory moderate, C – sensory severe). The CT-difference profiles were obtained from our LEAP data, and the expression profiles from the Allen Human Brian Atlas (AHBA), in our glutamate-pathway and GABA-pathway gene-sets. The x-axes show the correlation coefficient between CT and expression profile for all genes in the gene-set; the y-axes show the estimated probability density for the correlation coefficients; the vertical dashed-lines indicates the average expression-CT correlation coefficient across all the marker genes in a gene-set; and the edges of the gray boxes indicates the 2.5% and 97.5%-critical values obtained from the empirical null distribution of the average expression-thickness correlation coefficient. If a vertical line sits outside the gray box, it implies that there is a significant association between gene-set and CT profiles at the unadjusted 5% significance level.

**References**

1. Ecker C, Pretzsch CM, Jones EJH, Leap Team T, Murphy D. Inter-individual differences in cortical thickness and their genomic underpinnings in autism spectrum disorder. American Journal of Psychiatry. 2021;

2. Martino AD, Castellanos FX, Assaf M, Deen B. The Autism Brain Imaging Data Exchange: Towards Large-Scale Evaluation of the Intrinsic Brain Architecture in Autism. 2014;20.

3. Tillmann J, Uljarevic M, Crawley D, Dumas G, Loth E, Murphy D, et al. Dissecting the phenotypic heterogeneity in sensory features in autism spectrum disorder: a factor mixture modelling approach. Molecular Autism. 2020 Dec;11(1):67.
